# Artificial intelligence literacy, artificial intelligence anxiety, and career optimism among university music students: a cross-sectional study

**DOI:** 10.3389/fpsyg.2026.1961896

**Published:** 2026-10-16

**Authors:** Yang Du

**Affiliations:** School of Music and Dance, Xihua University, Chengdu, China

**Keywords:** AI education, artificial intelligence anxiety, artificial intelligence literacy, career development, career optimism, university music students

## Abstract

**Objective:**

To examine the levels of artificial intelligence (AI) literacy, AI anxiety, and career optimism among university music students, their associations, and the independent associations of AI literacy and AI anxiety with career optimism after adjustment for relevant background factors.

**Methods:**

A cross-sectional survey was conducted from May to July 2026 among students from five undergraduate music institutions in eastern China using convenience sampling. A total of 453 valid questionnaires were included in the analysis. Participants completed the Artificial Intelligence Literacy Scale for Chinese College Students (AILS-CCS), the Chinese version of the Artificial Intelligence Anxiety Scale (AIAS), and the Career Optimism Subscale (COS). Data were analyzed using descriptive statistics, independent-samples *t tests*, one-way analysis of variance, Pearson correlation analysis, and hierarchical multiple linear regression.

**Results:**

Mean scores for AI literacy, AI anxiety, and career optimism were 52.97 ± 10.05, 83.85 ± 22.96, and 37.87 ± 8.02, respectively. Students with prior AI training and those reporting more frequent AI use showed higher AI literacy and career optimism and lower AI anxiety. AI literacy was positively associated with career optimism (r = 0.444, *p* < 0.001), whereas AI anxiety was negatively associated with career optimism (r = −0.461, *p* < 0.001). After adjustment for gender, year of study, music specialization, prior AI training, and frequency of AI use, AI literacy remained positively associated with career optimism (β = 0.314, *p* < 0.001), whereas AI anxiety remained negatively associated with career optimism (β = −0.326, *p* < 0.001). The final model explained 33.0% of the variance in career optimism.

**Conclusion:**

AI literacy and AI anxiety were independently associated with career optimism among university music students. These findings suggest that students' perceptions of AI-related competence and technological concerns are relevant to their career expectations. Music institutions may consider AI education that combines practical competence with critical understanding of AI's implications for artistic identity, professional value, and future career development. Future longitudinal research is needed to clarify the temporal relationships among AI literacy, AI anxiety, and career optimism.

## 1 Introduction

Artificial intelligence (AI) is reshaping the disciplinary landscape of higher education and approaches to student education and workforce preparation at an unprecedented pace. A survey of more than 3,800 university students across 16 countries found that 86% of respondents used AI for academic purposes, with more than half reporting use at least once per week. However, 58% reported insufficient AI-related knowledge and skills, and 48% felt unprepared for an AI-driven labor market (Digital Education Council, 2024). This gap between widespread adoption and perceived competence suggests that the integration of AI into higher education may be advancing faster than students' capacity to use it effectively and critically. At the same time, universities are moving from ad hoc responses toward more formalized institutional governance of AI. A 2025 UNESCO survey of 400 respondents from UNESCO Chairs and UNITWIN Networks across 90 countries found that 19% reported that their institutions had established formal AI policies, whereas 42% indicated that such guidelines were still under development (UNESCO, 2025). These institutional changes are occurring alongside broader AI-driven changes in the labor market. Notably, the International Labor Organization estimated that approximately one quarter of workers worldwide are employed in occupations exposed to generative AI to varying degrees, while emphasizing that AI is more likely to transform tasks and skill requirements within occupations than to replace entire occupations (Gmyrek et al., 2025).

These changes are particularly salient for university music students. AI has already become integrated into a wide range of music-related practices, including music generation, arrangement, sound synthesis, audio processing, accompaniment production, and instructional feedback (Wang, 2025, 2026). Although AI technologies have lowered barriers to music creation, they have also raised fundamental concerns about originality, cultural bias, copyright, and artistic agency (Drury, 2025; Loor Paredes, 2025). Cheng (2025), for example, noted that generative AI has expanded creative possibilities in music education while also creating practical challenges concerning originality, cultural bias, equity, and ethical practice. Undergraduate music students are at a critical stage of professional skill development and career identity formation. For these students, AI therefore has a dual role: it can broaden learning opportunities and support the development of career-relevant capabilities, yet its growing involvement in core musical activities may also challenge students' perceptions of the distinctive value of their expertise and their future place in the profession.

AI literacy and AI anxiety provide two interrelated yet conceptually distinct frameworks for understanding music students' cognitive and emotional responses to technological change. AI literacy refers to an integrated set of competencies that enables individuals to understand AI, use AI tools effectively, critically evaluate AI-generated content, and assess its ethical and social implications (Ng et al., 2021; Stolpe and Hallström, 2024). Wang et al. (2023) identified four core dimensions of AI literacy: awareness, use, evaluation, and ethics. For music students, AI literacy extends beyond technical proficiency in using AI tools. It also encompasses the ability to recognize the appropriate scope and limitations of AI in music learning and creation, evaluate the artistic quality of AI-generated content, and critically examine issues related to originality, copyright, and AI ethics. Recent research among music students suggests that AI readiness is associated with self-efficacy and academic performance, indicating that AI-related competence may facilitate students' adaptation to AI-integrated learning environments (Wang and Li, 2024). In this sense, AI literacy represents a multidimensional competence that integrates technological understanding, practical application, critical evaluation, and ethical judgment. By contrast, AI anxiety refers to feelings of apprehension and unease arising from engagement with AI and perceptions of its potential risks. Wang and Wang (2022) conceptualized AI anxiety as comprising learning anxiety, job replacement anxiety, sociotechnical blindness, and AI configuration anxiety. AI literacy and AI anxiety thus capture distinct aspects of students' adaptation to AI environments: the former reflects their capacity to understand and engage with AI, whereas the latter reflects their perceived threat from it. According to Cognitive Appraisal Theory (CAT), the same technological change may be appraised as either a manageable challenge that facilitates growth or a threat that exceeds perceived coping resources (Lazarus and Folkman, 1984). Students' perceptions of the career implications of AI may therefore be shaped by the relative balance between challenge and threat appraisals.

Emerging evidence suggests that cognitive and emotional responses to AI coexist in complex ways among music students. Ouyang (2025) found that advances in AI voice technologies were associated with concerns about artistic identity and career development among vocal performance students. AI anxiety was negatively associated with externally evaluated artistic expressiveness, and students with higher levels of anxiety tended to underestimate their own artistic performance. Tan et al. (2025) further found that undergraduate music students recognized the educational benefits of anthropomorphic AI-generated content features, although stronger anthropomorphic characteristics were also associated with psychological discomfort. Li (2026) found enjoyment mediated the link between smart service interactional experience and behavioral intention among Chinese music students, underscoring positive emotions in GenAI music learning. Together, these findings indicate that music students' experiences of AI cannot be reduced to simple “acceptance” or “rejection.” Rather, perceived technological empowerment may coexist with concerns about professional identity and expertise. As AI becomes increasingly embedded in domains traditionally associated with human creativity, including composition, sound generation, and artistic expression, students may reassess their artistic identity, professional value, and career prospects. These responses extend beyond conventional technology acceptance because they are closely tied to creative agency, artistic identity, and perceptions of the distinctive value of musical expertise.

These responses raise a central question: how students perceive their future career prospects. Career optimism refers to positive expectations about future career development and confidence in achieving career goals. Previous research has linked higher career optimism to greater engagement in career planning and more positive career-related expectations and cognitions (Rottinghaus et al., 2005; Eva et al., 2020). As AI reshapes occupational tasks and skill requirements, students' perceived capacity to adapt to AI and their perceptions of AI-related career threats may be closely associated with career optimism. Recent evidence indicates that AI anxiety is associated with career-related outcomes, including college students' career decisions and career adaptability (Duan et al., 2026). According to Social Cognitive Career Theory (SCCT), career development is shaped by self-efficacy, outcome expectations, personal goals, and perceived environmental supports and barriers (Lent et al., 1994). Accordingly, AI literacy, as a career-relevant competence for navigating technological change, and AI anxiety, as an emotional response to perceived technological threat, may both be closely associated with career optimism. In addition, AI readiness has been linked to career adaptability and career decision-making among higher education students, suggesting that AI-related capabilities may contribute to career development (Testa et al., 2026).

Previous research has provided preliminary support for these relationships. Li et al. (2025) found an association between career self-efficacy and job-search anxiety and reported significant mediating roles for attitudes toward AI and AI literacy. In the field of music education, Wei et al. (2025), in a survey of 624 undergraduate music education students from 66 institutions, found that AI use positively predicted academic performance. Academic self-efficacy and AI dependence mediated this relationship, whereas institutional AI readiness moderated it. However, these studies did not examine AI literacy, AI anxiety, and career optimism simultaneously within a single analytical framework. The most directly relevant evidence comes from Güler et al. (2026), who surveyed undergraduate students in physiotherapy and rehabilitation. AI literacy was moderately and positively correlated with career optimism (*r* = 0.396), whereas AI anxiety was weakly and negatively correlated with career optimism (*r* = −0.147). After adjustment for demographic characteristics and AI-use variables, AI literacy remained independently associated with career optimism, whereas AI anxiety was no longer independently associated with career optimism. Whether these findings extend to university music students remains unclear. Although physiotherapy and rehabilitation also rely heavily on interpersonal interaction and practical skills, music disciplines additionally involve creative expression, aesthetic judgment, performance practice, and artistic identity formation. Moreover, generative AI is becoming increasingly integrated into core musical activities, including music generation and creative production. It therefore remains unclear whether, among music students, AI anxiety is more strongly negatively associated with career optimism and whether the positive association between AI literacy and career optimism remains robust.

AI literacy and AI anxiety may also be interrelated. The complexity and uncertainty of emerging technologies may heighten perceived threat among individuals with limited knowledge, whereas a clearer understanding of AI mechanisms and limitations may support more confident and effective engagement. Existing evidence suggests a negative relationship between AI literacy and AI anxiety. For example, Güler et al. (2026) reported a correlation of −0.253 between these two variables. Among music students, however, AI anxiety may not arise solely from limited technological competence. Even students proficient in using AI tools may remain concerned about AI's implications for artistic originality, musicians' employment opportunities, and the continuing value of performance and creative skills developed through years of professional training. Higher AI literacy does not necessarily correspond to the absence of AI anxiety, and the two constructs should therefore not be regarded as opposite ends of a single psychological continuum. Examining AI literacy, AI anxiety, and their respective dimensions simultaneously may therefore provide a more comprehensive account of the interplay between AI-related competence and emotional responses among music students.

Taken together, the existing literature reveals three important gaps. First, research on music and AI has largely focused on instructional applications, technology acceptance, and ethical issues, with less attention to students' career-related psychological responses. In particular, AI literacy, AI anxiety, and career optimism have rarely been examined together among university music students. Second, although Güler et al. (2026) examined relationships among these three constructs, their sample consisted of physiotherapy and rehabilitation students. Given the distinctive characteristics of music training, including embodied skills, creative expression, and artistic identity formation, it remains unclear whether these associations are also evident among music students. Third, background factors such as gender, year of study, music specialization, frequency of AI use, and prior AI training may confound the observed associations among these variables. It therefore remains unclear whether AI literacy and AI anxiety are independently associated with career optimism after adjustment for these factors.

Accordingly, the present study examined university music students in China. The study aimed to address the limited evidence on career-related psychological responses to AI in music education and to inform AI literacy education and career support in music institutions. To integrate these perspectives, Figure 1 presents the conceptual model. Drawing on SCCT (Lent et al., 1994) and CAT (Lazarus and Folkman, 1984), AI literacy is viewed as a career-relevant capability, whereas AI anxiety is viewed as threat appraisal. The two constructs are conceptually distinct but negatively related, and both are expected to relate to career optimism. Covariates include gender, year of study, music specialization, prior AI training, and frequency of AI use. We hypothesize:

- H1: AI literacy is positively associated with career optimism among university music students.
- H2: AI anxiety is negatively associated with career optimism among university music students.
- H3: AI literacy is negatively associated with AI anxiety among university music students.
- H4: AI literacy and AI anxiety are independently associated with career optimism after adjustment for gender, year of study, music specialization, prior AI training, and frequency of AI use.

**Figure 1:**
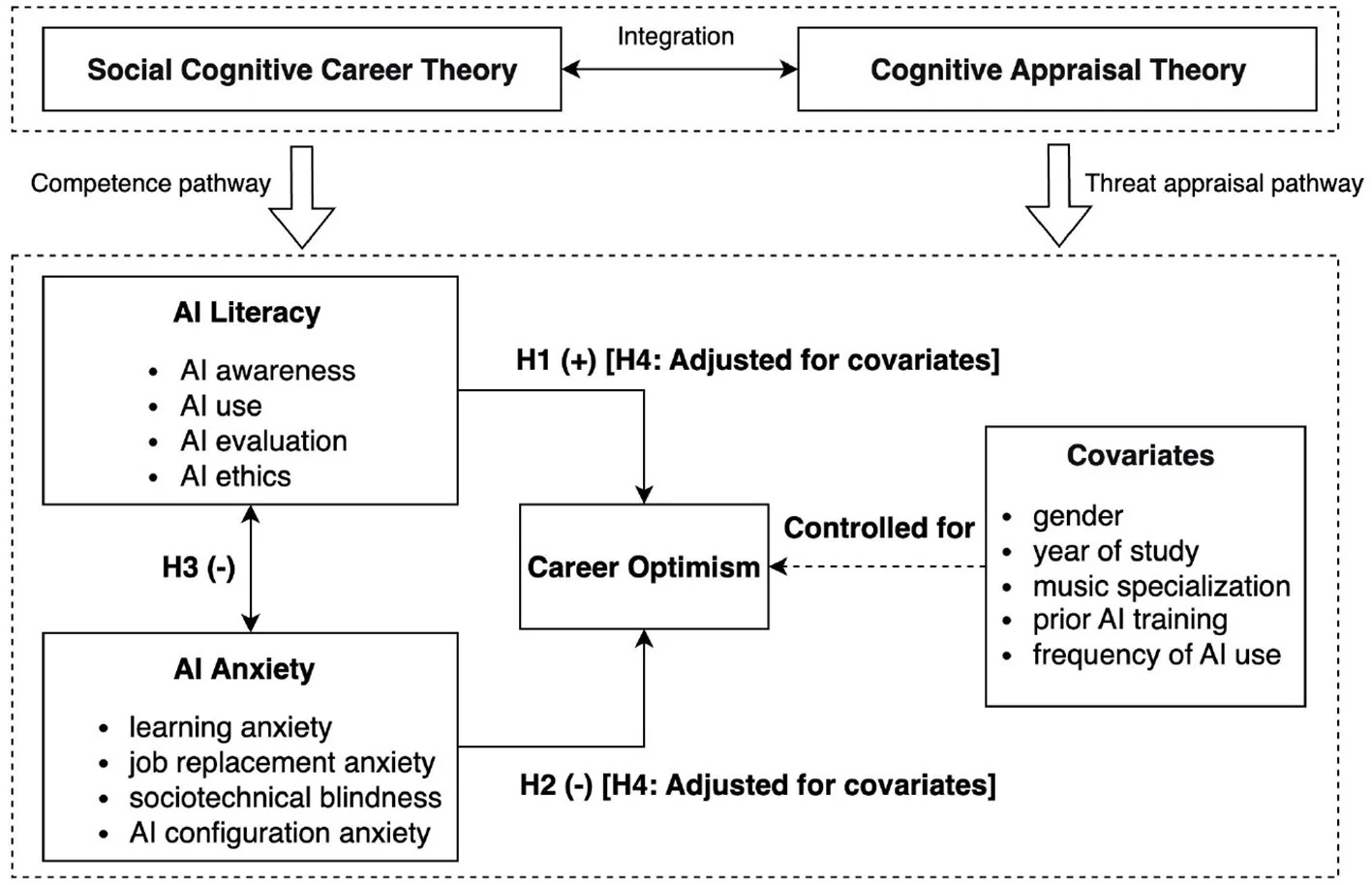
Conceptual model of hypothesized associations among AI literacy, AI anxiety, and career optimism. The model integrates SCCT (competence pathway) and CAT (threat appraisal pathway). H1: positive association; H2: negative association; H3: negative correlation; H4: associations of AI literacy and AI anxiety with career optimism after adjustment for covariates. Solid arrows indicate hypothesized associations, not causal effects; the dashed arrow indicates controlled covariates.

## 2 Methods

### 2.1 Study design

This study employed a cross-sectional survey design among students from five undergraduate music institutions in eastern China. Data were collected using an anonymous online questionnaire. The study aimed to examine levels of AI literacy, AI anxiety, and career optimism, the relationships among these variables, and their associations with demographic characteristics, music-related academic background, and AI-use experience.

### 2.2 Participants

A convenience sampling strategy was used, consistent with recent questionnaire-based research involving university music students (Xu and Li, 2025). From May to July 2026, students enrolled in music-related programs at five undergraduate music institutions in eastern China were recruited. Recruitment information was distributed through institutional online channels, including class WeChat groups and subject-specific learning groups. Participation was voluntary, and no names or other directly identifying information were collected.

The inclusion criteria were: (1) current enrollment in a music-related program at one of the five participating institutions; (2) age ≥18 years; (3) ability to independently read and complete the online questionnaire; and (4) provision of electronic informed consent. The exclusion criteria were: (1) enrollment in a non-music-related program; (2) incomplete questionnaire submission; and (3) responses identified as invalid during data-quality screening because of markedly abnormal completion times, patterned responding, logical inconsistencies, or suspected duplicate submissions.

The required sample size was estimated a priori using G^*^Power for the planned multiple linear regression analysis. Assuming a small effect size (Cohen's *f*^2^ = 0.05), an α level of 0.05, a statistical power of 0.80, and 10 predictors, the minimum required sample size was 335 participants. A total of 521 questionnaires were submitted during the survey period. After 68 questionnaires were excluded following completeness and data-quality screening, 453 were retained for analysis, representing 87.0% of all submitted questionnaires. The final sample therefore exceeded the a priori minimum sample-size requirement.

### 2.3 Measures

#### 2.3.1 Participant characteristics questionnaire

A researcher-developed questionnaire was used to collect participant characteristics. Information collected included gender, age, year of study, music specialization, prior AI-related training, and frequency of AI use.

#### 2.3.2 Artificial intelligence literacy scale

AI literacy was assessed using the Artificial intelligence Literacy Scale for Chinese College Students (AILS-CCS; Ma and Chen, 2024). The scale consists of 15 items across four dimensions: AI awareness (4 items), AI use (3 items), AI evaluation (4 items), and AI ethics (4 items). These dimensions assess students' understanding of basic AI concepts and principles, use of AI tools for learning and everyday problem solving, critical evaluation of AI tools and generated content, and ethical AI use, including privacy protection and recognition of misuse risks, respectively.

Each item is rated on a 5-point Likert scale ranging from 1 (“strongly disagree”) to 5 (“strongly agree”). All items are positively scored and summed to produce a total score ranging from 15 to 75, with higher scores indicating greater self-perceived AI literacy. Previous validation demonstrated good psychometric properties, with composite reliability coefficients of 0.849–0.890 and average variance extracted values of 0.587–0.676, together with evidence of construct, convergent, and discriminant validity (Ma and Chen, 2024). In the present study, Cronbach's α ranged from 0.838 to 0.861 across the four dimensions and was 0.928 for the total scale, while confirmatory factor analysis (CFA) showed an acceptable fit for the four-factor model (χ^2^/df = 2.74, CFI = 0.921, TLI = 0.907, RMSEA = 0.074, SRMR = 0.058), with CR and AVE values exceeding recommended thresholds and supporting satisfactory construct reliability and convergent validity.

#### 2.3.3 Artificial intelligence anxiety scale

AI anxiety was assessed using the Chinese version of the Artificial Intelligence Anxiety Scale (AIAS), adapted by Wang et al. (2026) from the original scale developed by Wang and Wang (2022). The scale contains 21 items across four dimensions: learning anxiety (8 items), job replacement anxiety (6 items), sociotechnical blindness (4 items), and AI configuration anxiety (3 items). These dimensions assess anxiety related to learning about and using AI, job replacement, perceived sociotechnical risks, and interaction with human-like AI systems, respectively.

Items are rated on a 7-point Likert scale ranging from 1 (“strongly disagree”) to 7 (“strongly agree”). Item scores are summed to yield a total score of 21–147, with higher scores indicating greater AI anxiety. The original scale demonstrated good internal consistency, with Cronbach's α = 0.964 for the total scale and values ranging from 0.917 to 0.974 across its dimensions (Wang and Wang, 2022). In the present study, Cronbach's α ranged from 0.836 to 0.917 across the four dimensions and was 0.952 for the total scale, while CFA supported the four-factor structure of the AI anxiety scale (χ^2^/df = 2.83, CFI = 0.918, TLI = 0.905, RMSEA = 0.076, SRMR = 0.061), with CR and AVE values meeting recommended criteria for construct reliability and convergent validity.

#### 2.3.4 Career optimism subscale

Career optimism was assessed using the Chinese version of the Career Optimism Subscale (COS) adapted by Yang (2023). The subscale was derived from the Career Futures Inventory (CFI) developed by Rottinghaus et al. (2005) and consists of 11 items. It assesses positive expectations about future career development, confidence in achieving career goals, and active engagement in career planning and decision-making.

Items are rated on a 5-point Likert scale ranging from 1 (“strongly disagree”) to 5 (“strongly agree”). The subscale contains both positively and negatively worded items. Negatively worded items are reverse-scored before scores are summed. Total scores range from 11 to 55, with higher scores indicating greater career optimism. The original subscale demonstrated good internal consistency (Cronbach's α = 0.87; Rottinghaus et al., 2005). In the present study, Cronbach's α was 0.890, and CFA indicated an acceptable one-factor model fit (χ^2^/df = 2.65, CFI = 0.931, TLI = 0.914, RMSEA = 0.071, SRMR = 0.054), with satisfactory construct reliability and convergent validity.

### 2.4 Data collection

The study was approved by the Ethics Committee of Xihua University (No. XHLL-2025-K062) and conducted in accordance with the Declaration of Helsinki. Faculty members, student counselors, or student representatives in relevant departments at the five participating institutions were contacted and informed of the study objectives, eligibility criteria, and survey procedures. After permission was obtained, institutional contacts distributed the study invitation and questionnaire link through class WeChat groups and subject-specific learning groups. The same recruitment materials, questionnaire, and data-collection procedures were used at all five institutions.

The questionnaire was administered via the Wenjuanxing online survey platform. The first page described the study purpose, anonymity, intended use of the data, voluntary nature of participation, and right to withdraw. The estimated completion time was 15–20 min. Participants could access the questionnaire only after providing electronic informed consent. After data collection, questionnaires were screened for completeness, completion time, response patterns, logical consistency, and duplicate submissions. After screening, reverse-scored items were recoded, variables were coded, and scale scores were verified before the final analytic dataset was established.

### 2.5 Statistical analysis

Data were analyzed using IBM SPSS Statistics, version 27.0. Participant characteristics and study variables were summarized using frequencies, percentages, means, and standard deviations, and internal consistency was assessed using Cronbach's α. Confirmatory factor analysis (CFA) was conducted to evaluate construct validity, and composite reliability (CR) and average variance extracted (AVE) were calculated to assess reliability and convergent validity. Harman's single-factor test was performed to assess potential common method bias. Pearson correlation coefficients were calculated to examine associations among the primary study variables. Independent-samples *t* tests were used for two-group comparisons, and one-way analysis of variance (ANOVA) was used for variables with more than two groups. When the homogeneity-of-variance assumption was violated, Welch's ANOVA was used. When an omnibus test was statistically significant, Bonferroni-adjusted pairwise comparisons were performed.

Hierarchical multiple linear regression was performed with career optimism total score as the dependent variable. Model 1 included total AI literacy and AI anxiety scores. Model 2 additionally included gender, year of study, music specialization, frequency of AI use, and prior AI training as covariates. Categorical variables were dummy coded, with female gender, first-year students, instrumental performance, and no prior AI training serving as reference categories.

Before regression analysis, linearity, residual normality, homoscedasticity, multicollinearity, and influential observations were assessed. Multicollinearity was evaluated using tolerance and variance inflation factor (VIF) values. Given the cross-sectional design, regression findings were interpreted as statistical associations rather than causal effects. All tests were two-sided, with p < 0.05 considered statistically significant.

## 3 Results

### 3.1 Participant characteristics

A total of 453 university music students were included in the analysis. The largest age group was 20–21 years (43.0%), and 67.5% of participants were female. Participants were distributed relatively evenly across the five institutions and the 4 years of study. Instrumental performance (33.3%) and music education (26.0%) were the most common specializations. Approximately one third of participants had received AI-related training (32.9%). Regarding AI-use frequency, 39.1% of participants reported using AI 3–5 times per day, whereas 35.3% reported using it ≥6 times per day. Detailed demographic and AI-use characteristics are presented in Table 1.

**Table 1:** Sociodemographic and AI-related characteristics of participants.

Variable	Category	*n*	Percentage (%)
Age	18-19 years	148	32.7
	20-21 years	195	43
	22-23 years	110	24.3
Institution	Institution A	106	23.4
	Institution B	91	20.1
	Institution C	74	16.3
	Institution D	88	19.4
	Institution E	94	20.8
Gender	Female	306	67.5
	Male	147	32.5
Year of study	First year	120	26.5
	Second year	118	26
	Third year	112	24.7
	Fourth year	103	22.7
Music specialization	Instrumental performance	151	33.3
	Vocal performance	92	20.3
	Music education	118	26
	Composition and music theory	45	9.9
	Music technology and production	47	10.4
Prior AI-related training	No	304	67.1
	Yes	149	32.9
Frequency of AI use	≤ requency of day	116	25.6
	35.6uency of Aday	177	39.1
	≥9.1uency of day	160	35.3

### 3.2 Descriptive statistics of the main study variables

The mean AI literacy score was 52.97 ± 10.05, with a range of 16–75 and a mean item score of 3.53. Among the AI literacy dimensions, AI ethics had the highest mean item score (3.70), whereas AI use had the lowest (3.38). The mean AI anxiety score was 83.85 ± 22.96, with a range of 26–144 and a mean item score of 3.99. Among the AI anxiety dimensions, job replacement anxiety had the highest mean item score (4.37), whereas learning anxiety had the lowest (3.74). The mean career optimism score was 37.87 ± 8.02, with a range of 14–55 and a mean item score of 3.44. Detailed descriptive statistics are presented in Table 2.

**Table 2:** Descriptive statistics of the main study variables.

Variable	Minimum	Maximum	Mean ±SD	Mean item score
Total AI literacy	16	75	52.97 AI lite	3.53
AI awareness	4	20	14.19 reness	3.55
AI use	3	15	10.15 reness	3.38
AI evaluation	4	20	13.82 luati9	3.46
AI ethics	5	20	14.80 icsti9	3.7
Total AI anxiety	26	144	83.85 AI anxi	3.99
Learning anxiety	8	56	29.92 ng anx	3.74
Job replacement anxiety	7	42	26.20 placem	4.37
Sociotechnical blindness	4	28	16.08 echnic	4.02
AI configuration anxiety	3	21	11.65 ± 4.11	3.88
Total career optimism	14	55	37.87 career	3.44

### 3.3 Group differences in the main study variables

Independent-samples *t tests* showed significant differences in AI literacy, AI anxiety, and career optimism between students with and without prior AI-related training (see Table 3). Students with prior AI training had significantly higher AI literacy scores than those without training (56.66 ± 8.69 vs. 51.16 ± 10.20), *t* (451) = −5.655, *p* < 0.001, Cohen's *d* = 0.565. They also had significantly lower AI anxiety scores (77.58 ± 21.48 vs. 86.93 ± 23.07), *t* (451) = 4.144, *p* < 0.001, Cohen's d = 0.414, and higher career optimism scores (40.25 ± 8.08 vs. 36.70 ± 7.74), *t* (451) = −4.511, *p* < 0.001, Cohen's *d* = 0.451.

**Table 3:** Group differences in the main study variables.

Grouping Variable	Category	*n*	AI Literacy M ±SD	*t/F*	*p*	AI Anxiety M ±SD	*t/F*	*p*	Career Optimism M ±SD	*t/F*	*p*
AI training experience	No prior training	304	51.16 ± 10.20	−5.655	< 0.001	86.93 ± 23.07	4.144	< 0.001	36.70 ± 7.74	−4.511	< 0.001
	Received training	149	56.66 ± 8.69			77.58 ± 21.48			40.25 ± 8.08		
Year of study	First year	120	51.86 ± 8.63	1.642^a^	0.18	82.06 ± 21.68	1.309	0.271	38.52 ± 7.91	0.366	0.778
	Second year	118	52.81 ± 11.16			82.23 ± 21.83			37.56 ± 8.49		
	Third year	112	52.85 ± 10.67			84.14 ± 24.45			37.76 ± 7.28		
	Fourth year	103	54.57 ± 9.50			87.49 ± 23.88			37.59 ± 8.44		
Music specialization	Instrumental performance	151	52.03 ± 10.16	2.379	0.051	88.52 ± 23.04	3.609	0.007	37.05 ± 7.84	2.759	0.027
	Vocal performance	92	53.47 ± 10.56			83.45 ± 20.55			36.46 ± 7.79		
	Music education	118	52.20 ± 9.30			83.32 ± 23.80			38.52 ± 8.33		
	Composition and music theory	45	53.00 ± 9.39			76.04 ± 24.12			39.33 ± 8.62		
	Music technology and production	47	56.89 ± 10.55			78.47 ± 21.33			40.26 ± 7.01		
Frequency of AI use	≤ 2 times per day	116	48.53 ± 10.57	20.69	< 0.001	93.09 ± 23.78	18.858	< 0.001	35.04 ± 7.75	17.122	< 0.001
	3–5 times per day	177	53.06 ± 8.83			84.38 ± 21.80			37.37 ± 7.87		
	≥6 times per day	160	56.09 ± 9.80			76.57 ± 21.16			40.48 ± 7.62		

*N* = 453. Data are presented as M ± SD. Independent-samples t tests were used for comparisons by AI training experience, whereas one-way analysis of variance (ANOVA) was used for the other grouping variables. ^a^The homogeneity-of-variance assumption was violated for AI literacy across years of study; therefore, Welch's ANOVA was used, Welch's *F* (3, 246.476) = 1.642, *p* = 0.180. All other *F* values were obtained from standard one-way ANOVA. For significant omnibus tests, Bonferroni-adjusted pairwise comparisons were conducted. Bold values indicate statistical significance at *p* < 0.05.

No statistically significant differences were observed by year of study for any of the three main variables. Because the homogeneity-of-variance assumption was violated for AI literacy, Welch's ANOVA was conducted and showed no significant difference by year of study, Welch's *F* (3, 246.476) = 1.642, *p* = 0.180. Differences in AI anxiety and career optimism by year of study were also non-significant, *F* (3, 449) = 1.309, *p* = 0.271, η^2^ = 0.009, and *F* (3, 449) = 0.366, *p* = 0.778, η^2^ = 0.002, respectively.

AI literacy did not differ significantly across music specializations, *F* (4, 448) = 2.379, *p* = 0.051, η^2^ = 0.021. In contrast, AI anxiety differed significantly across music specializations, *F* (4, 448) = 3.609, *p* = 0.007, η^2^ = 0.031. Bonferroni-adjusted pairwise comparisons showed that instrumental performance students had higher AI anxiety scores than students in composition and music theory (88.52 ± 23.04 vs. 76.04 ± 24.12, *p* = 0.013). Career optimism also showed a significant omnibus difference across music specializations, *F* (4, 448) = 2.759, *p* = 0.027, η^2^ = 0.024. However, after Bonferroni correction, no pairwise comparison between specializations was statistically significant.

All three main variables differed significantly across AI-use frequency groups. Mean AI literacy scores were 48.53 ± 10.57, 53.06 ± 8.83, and 56.09 ± 9.80 for students using AI ≤ 2 times per day, 3–5 times per day, and ≥6 times per day, respectively, *F* (2, 450) = 20.690, *p* < 0.001, η^2^ = 0.084. Corresponding mean AI anxiety scores were 93.09 ± 23.78, 84.38 ± 21.80, and 76.57 ± 21.16, *F* (2, 450) = 18.858, *p* < 0.001, η^2^ = 0.077. Mean career optimism scores were 35.04 ± 7.75, 37.37 ± 7.87, and 40.48 ± 7.62, respectively, *F* (2, 450) = 17.122, *p* < 0.001, η^2^ = 0.071. Bonferroni-adjusted pairwise comparisons showed that each AI-use frequency group differed significantly from the other two groups on all three variables.

Institutional differences were also examined using one-way ANOVA. No significant differences were observed across the five institutions in AI literacy, AI anxiety, or career optimism (all *p* > 0.05). Therefore, institution was not included as an additional covariate in the hierarchical regression models.

### 3.4 Correlations among AI literacy, AI anxiety, and career optimism

Pearson correlation analysis showed significant associations among AI literacy, AI anxiety, and career optimism. AI literacy was negatively correlated with AI anxiety (*r* = −0.321, *p* < 0.001) and positively correlated with career optimism (*r* = 0.444, *p* < 0.001). AI anxiety was negatively correlated with career optimism (*r* = −0.461, *p* < 0.001).

At the dimension level, AI awareness, AI use, AI evaluation, and AI ethics were positively correlated with career optimism, with *r* values of 0.378, 0.391, 0.333, and 0.390, respectively (all *p* < 0.001). AI use had the largest positive correlation coefficient with career optimism (*r* = 0.391), although this was nearly identical to that for AI ethics (*r* = 0.390). Learning anxiety, job replacement anxiety, sociotechnical blindness, and AI configuration anxiety were negatively correlated with career optimism, with *r* values of −0.413, −0.399, −0.364, and −0.444, respectively (all *p* < 0.001). AI configuration anxiety showed the largest negative correlation in absolute magnitude with career optimism (*r* = −0.444).

Overall, higher AI literacy scores, both overall and across dimensions, were associated with greater career optimism, whereas higher AI anxiety scores, both overall and across dimensions, were associated with lower career optimism. Detailed correlation results are presented in Tables 4, 5.

**Table 4:** Correlations among the three main study variables.

Variable	Mean	SD	1	2	3
1. AI literacy	52.97	10.05	1		
2. AI anxiety	83.85	22.96	−0.321^***^	1	
3. Career optimism	37.87	8.02	0.444^***^	−0.461^***^	1

^***^ *p* < 0.001, two-tailed.

**Table 5:** Correlations between the dimensions of AI literacy and AI Anxiety and career optimism.

Variable	*r* with career optimism
AI awareness	0.378^***^
AI use	0.391^***^
AI evaluation	0.333^***^
AI ethics	0.390^***^
Learning anxiety	−0.413^***^
Job replacement anxiety	−0.399^***^
Sociotechnical blindness	−0.364^***^
AI configuration anxiety	−0.444^***^

*N* = 453. ^***^ *p* < 0.001, two-tailed.

A Harman's single-factor test was conducted to assess potential common method bias arising from the use of self-report measures. The first factor accounted for 28.64% of the total variance, which was below the recommended threshold of 40%, suggesting that common method bias was unlikely to substantially influence the results.

### 3.5 Hierarchical multiple regression predicting career optimism

Hierarchical multiple linear regression was conducted with the total career optimism score as the dependent variable (Table 6). In Model 1, total AI literacy and AI anxiety scores were entered simultaneously. Model 1 was statistically significant, *F* (2, 450) = 101.059, *p* < 0.001, and explained 31.0% of the variance in career optimism (*R*^2^ = 0.310, adjusted *R*^2^ = 0.307). AI literacy was positively associated with career optimism (*B* = 0.263, SE = 0.033, β = 0.330, *t* = 7.981, *p* < 0.001, 95% CI [0.198, 0.328]), whereas AI anxiety was negatively associated with career optimism (*B* = −0.124, SE = 0.014, β = −0.355, *t* = −8.587, *p* < 0.001, 95% CI [−0.152, −0.096]).

**Table 6:** Hierarchical multiple linear regression analysis predicting career optimism.

Predictor	Model 1 B (SE)	β	*p*	95% CI	Model 2 B (SE)	β	*p*	95% CI
Constant	34.325 (2.445)	—	< 0.001	[29.521, 39.129]	33.143 (2.675)	—	< 0.001	[27.887, 38.400]
AI literacy	0.263 (0.033)	0.33	< 0.001	[0.198, 0.328]	0.250 (0.034)	0.314	< 0.001	[0.183, 0.318]
AI anxiety	−0.124 (0.014)	−0.355	< 0.001	[−0.152, −0.096]	−0.114 (0.015)	−0.326	< 0.001	[−0.143, −0.084]
Gender: male	—	—	—	—	0.067 (0.690)	0.004	0.923	[−1.289, 1.422]
Year of study	—	—	—	—	−0.328 (0.291)	−0.045	0.26	[−0.901, 0.244]
Vocal performance	—	—	—	—	−1.381 (0.893)	−0.069	0.123	[−3.136, 0.374]
Music education	—	—	—	—	0.897 (0.831)	0.049	0.281	[−0.737, 2.531]
Composition and music theory	—	—	—	—	0.394 (1.145)	0.015	0.731	[−1.855, 2.644]
Music technology and production	—	—	—	—	0.489 (1.134)	0.019	0.666	[−1.739, 2.717]
Prior AI training: yes	—	—	—	—	0.748 (0.737)	0.044	0.31	[−0.700, 2.197]
Frequency of AI use	—	—	—	—	0.710 (0.452)	0.069	0.117	[−0.178, 1.597]
R^2^	0.31				0.33			
Adjusted R^2^	0.307				0.314			
ΔR^2^	—				0.02			
F	101.059^***^				21.723^***^			
ΔF	—				1.614			

The dependent variable was the career optimism total score. Model 1 included total AI literacy and AI anxiety scores. Model 2 additionally included gender, year of study, music specialization, prior AI training, and frequency of AI use as covariates. Female, instrumental performance, and no prior AI training served as the reference categories for gender, music specialization, and AI training, respectively. Year of study was coded 1–4, and frequency of AI use was coded 1–3. *B* = unstandardized regression coefficient; SE = standard error; β = standardized regression coefficient; CI = confidence interval. ^***^ *p* < 0.001; — = not applicable.

Model 2 additionally included gender, year of study, music specialization, frequency of AI use, and prior AI training as covariates. Model 2 remained statistically significant, *F* (10, 442) = 21.723, *p* < 0.001, and explained 33.0% of the variance in career optimism (*R*^2^ = 0.330, adjusted *R*^2^ = 0.314). The covariates added in Model 2 accounted for an additional 2.0% of the variance in career optimism, but this increase was not statistically significant (Δ*R*^2^ = 0.020, Δ*F* (8, 442) = 1.614, *p* = 0.118).

After adjustment for these covariates, AI literacy remained positively associated with career optimism (*B* = 0.250, SE = 0.034, β = 0.314, *t* = 7.305, *p* < 0.001, 95% CI [0.183, 0.318]), whereas AI anxiety remained negatively associated with career optimism (*B* = −0.114, SE = 0.015, β = −0.326, *t* = −7.556, *p* < 0.001, 95% CI [−0.143, −0.084]). The standardized coefficients were similar in magnitude, although the absolute coefficient for AI anxiety was slightly larger than that for AI literacy (|β| = 0.326 vs. 0.314). None of the covariates—gender, year of study, music specialization, prior AI training, or frequency of AI use—was statistically significant in Model 2 (all *p* > 0.05).

## 4 Discussion

### 4.1 Coexistence of technological preparedness and career uncertainty

The present findings suggest that AI-related competence and career uncertainty may coexist among music students. Students with higher AI literacy reported greater preparedness for engaging with AI technologies, while concerns regarding technological substitution, professional value, and future career opportunities remained present. CAT suggests that individuals' responses to technological change depend on their evaluation of external demands and available coping resources. In the context of AI adoption, students may develop positive evaluations of AI when they perceive sufficient capability and resources to manage technological changes. At the same time, concerns related to employment, artistic identity, and professional development may be associated with stronger perceptions of technological threat.

This interpretation is consistent with Lintner's (2024) conceptualization of AI literacy as a multidimensional competence involving AI application skills, awareness of technological limitations, and understanding of broader social implications. Previous research has associated higher AI literacy with lower technology-related anxiety (Cengiz and Peker, 2025). The present findings extend this perspective by suggesting that AI literacy and AI anxiety may reflect different psychological responses during students' adaptation to AI development. AI literacy represents students' capability to understand, evaluate, and use AI technologies, while AI anxiety reflects concerns regarding potential changes brought by AI adoption. Therefore, students with stronger AI literacy may report greater confidence in engaging with AI while still experiencing uncertainty about future career transformation.

For music students, AI anxiety may involve concerns related to artistic identity, professional uniqueness, and career development. Ouyang (2025) identified concerns regarding artistic expression, identity vulnerability, and career uncertainty among vocal performance students, while Tan et al. (2025) reported that students recognized the educational value of AI while maintaining concerns about AI-related changes. Music training emphasizes long-term practice, embodied skills, individual expression, and emotional communication. These characteristics may increase students' attention to changes in the relationship between technology and professional expertise. Even students with greater AI competence may remain concerned about how AI-generated content and automated creative processes could influence perceptions of personal style and professional value (Ou et al., 2025). This perspective helps illustrate why AI-related competence and threat appraisal may occur simultaneously during students' adjustment to AI-related career changes.

### 4.2 Relationships of AI literacy and AI anxiety with career optimism

A growing body of research has linked AI literacy with more positive expectations regarding future learning and career development. Ma et al. (2025) reported associations among AI literacy, perceived career control, positive expectations, and career optimism, while Li et al. (2025)highlighted the role of AI literacy in managing uncertainty during the job-search process. Consistent with previous findings, AI literacy and all four of its dimensions were positively associated with career optimism in the present study. This association can be interpreted through SCCT, which emphasizes self-efficacy and outcome expectations as important factors in career development (Lent et al., 1994). Within this framework, AI literacy represents a technology-related career capability that may strengthen students' confidence in managing AI-related tasks and shape their expectations regarding future career opportunities. Among music students, AI literacy may support repertoire analysis, practice feedback, arranging, composition, and instructional design. The associations of AI awareness, AI use, AI evaluation, and AI ethics with career optimism suggest that students' career confidence may relate to their technological competence, professional judgment, and perceived ability to adapt to AI-related changes. These findings are consistent with Güler et al. (2026), who reported a positive association between AI literacy and career optimism among physiotherapy and rehabilitation students.

AI anxiety and all four of its dimensions were negatively associated with career optimism. This relationship can be interpreted through CAT (Lazarus and Folkman, 1984), which emphasizes individuals' evaluation of environmental demands and coping resources. In the context of AI adoption, students may experience anxiety when AI-related changes are perceived as difficult to manage. Job replacement anxiety may reflect concerns about employment opportunities and the future value of professional expertise, whereas learning anxiety may reflect concerns about acquiring AI-related knowledge and skills. These threat appraisals may be associated with lower perceived control over career development and lower career optimism. Duan et al. (2026) reported that AI anxiety was associated with lower career decision-making clarity and greater career indecision through reduced career adaptability, while Uçar et al. (2025) identified an association between AI anxiety and unemployment anxiety. For music students, these concerns may be intensified because AI increasingly interacts with creative production, artistic expression, and professional identity. The mechanisms proposed here were not directly tested in the present study and should be examined in future research.

Previous findings regarding AI anxiety and career outcomes have shown variation across disciplines. Yaşar and Karagucuk (2025) found no significant association between AI anxiety and career certainty among English education and literature students, while Güler et al. (2026) reported a weak negative correlation between AI anxiety and career optimism. Such differences may reflect variations in how closely AI is connected with core professional activities across disciplines. AI literacy and AI anxiety represent important psychological factors associated with career optimism, while career adaptability, professional identity, institutional support, and labor-market expectations may also influence students' career attitudes.

### 4.3 Adjusted associations of AI literacy and AI anxiety with career optimism

Hierarchical regression analysis showed that AI literacy and AI anxiety remained significantly associated with career optimism after adjustment for gender, year of study, music specialization, frequency of AI use, and prior AI training. These findings suggest that AI-related psychological factors provide additional explanatory value beyond demographic characteristics and AI-use experiences. Consistent with SCCT and CAT, AI literacy and AI anxiety may be associated with career optimism through different psychological pathways. AI literacy reflects students' perceived capability to engage with AI technologies, whereas AI anxiety reflects concerns regarding AI-related threats and coping demands. According to SCCT, perceived capability may influence career optimism through enhanced self-efficacy and more positive outcome expectations. Li et al. (2025) reported associations among career self-efficacy, AI attitudes, AI literacy, and job-search anxiety, while Duan et al. (2026) identified career adaptability as a potential mechanism linking AI anxiety with career decision-making.

The present findings differ partly from those of Güler et al. (2026). In their study, AI literacy remained independently associated with career optimism, whereas AI anxiety was no longer significant after adjustment. In contrast, AI anxiety showed an independent negative association with career optimism in the present study. This difference may relate to disciplinary characteristics. For music students, generative AI is closely connected with creative production, performance practices, and artistic identity. Performance-oriented students may be more concerned about changes in professional uniqueness, while technology-oriented students may perceive AI as a tool supporting creative activities.

Prior AI training and frequency of AI use were associated with career optimism in group comparisons, but neither remained significant after AI literacy and AI anxiety were included in the regression model. This finding suggests that frequency of AI use alone may have limited explanatory value for career optimism. Frequency of AI use reflects the amount of exposure to AI but does not necessarily capture the purpose, quality, dependence level, or patterns of human–AI collaboration. Students may use AI frequently for different purposes, including music learning, creative production, performance practice, or academic tasks, which may lead to different psychological experiences and career perceptions (Granström and Oppi, 2025). Future research should further examine specific contexts and patterns of AI use.

The final model explained 33.0% of the variance in career optimism, indicating that other individual and contextual factors may also contribute to students' career expectations. Future research should consider factors such as artistic self-efficacy, professional identity, family socioeconomic background, perceptions of the music industry, individual performance or composition skills, and institutional support.

### 4.4 Implications for AI education strategies in music institutions

Given the observed positive association between AI literacy and career optimism and the negative association between AI anxiety and career optimism, music institutions may benefit from integrating AI education more systematically into music training and career preparation. Consistent with evidence that AI readiness among music students is associated with self-efficacy and academic performance (Li, 2026), higher education research has emphasized the importance of developing students' AI literacy to support effective engagement with AI-driven learning and professional environments (Laupichler et al., 2022). AI education initiatives should therefore emphasize students' ability to use, evaluate, and critically reflect on AI technologies within authentic music contexts. Evidence from Chinese university music education indicates that teacher scaffolding plays an important role in supporting students' learning processes and discipline-specific development, highlighting the need to maintain instructor-guided learning within AI-enhanced educational contexts (Li and Wang, 2026). Composition and music production programs could use human–AI collaborative projects that require students to evaluate generated alternatives, justify revisions, and maintain responsibility for aesthetic decisions. Music education programs could emphasize AI-generated teaching materials, personalized feedback, and ethical issues related to learner data. Generative AI creates new opportunities for music creation and teaching but also raises concerns involving originality, copyright, cultural bias, and educational equity (Cheng, 2025). A human-centered approach may position AI as a tool that supports students' judgment, creativity, and professional agency. The goal of AI education should extend beyond increasing familiarity with AI tools to developing students' capacity to make informed decisions about when and how AI can be integrated into professional activities. Faculty development should address AI-informed task design, evaluation of AI-generated outputs, copyright and ethical issues, and students' concerns regarding AI-related career changes. Sustained coursework, practical workshops, and reflective activities may provide students with opportunities to connect AI experiences with professional competence and career adaptation (Møgelvang et al., 2025).

AI education should also consider differences across music disciplines and coordinate with assessment practices and institutional guidance. Students in different music fields may encounter different AI-related challenges. Performance-oriented students may focus more on artistic identity, embodied skills, and concerns about professional uniqueness, while students in music technology-related fields may engage with AI more frequently as a creative resource. When AI contributes to creative production, the final product alone may provide limited evidence of students' own analysis, selection, and revision processes. Assessment could therefore incorporate drafts, version histories, revision rationales, and oral explanations to make students' individual contributions more visible. Excessive reliance on AI may weaken independent judgment and critical engagement (Tian and Zhang, 2025), making it important to balance technological efficiency with aesthetic reasoning and creative decision-making. Institutions should also establish clear expectations for AI use in coursework, creative production, performance support, and academic writing, including guidance on copyright, data privacy, disclosure, and accountability (Jin et al., 2025). Such institutional support may reduce uncertainty related to AI use and provide clearer guidance for students as they evaluate future career development in AI-related environments.

### 4.5 Limitations and recommendations for future research

Several limitations should be acknowledged. First, the study used online convenience sampling and recruited participants from five undergraduate music institutions in eastern China, which may limit the generalizability of the findings to other regions and educational contexts. Music students also represent diverse professional groups, and students from different disciplines may have different perceptions of AI. Performance-oriented students may focus more on artistic identity and professional uniqueness, whereas students in music technology-related fields may engage with AI as a creative resource. Future research should examine AI-related perceptions across broader and more diverse samples.

Second, although Harman's single-factor test suggested that common method bias was unlikely to substantially influence the results, all core variables were assessed through self-report measures at the same time point. Therefore, subjective reporting bias cannot be completely excluded. In particular, the AI literacy scale measured perceived AI literacy rather than objectively demonstrated AI ability, and students' self-perceptions may differ from their actual AI-related performance. Future research could combine self-report measures with performance-based assessments and qualitative approaches.

Third, AI-use experience was primarily assessed through frequency of use, which may not fully capture differences in usage purpose, quality, dependence level, or patterns of human–AI collaboration. Students may use AI frequently for different purposes, including music learning, creative production, performance practice, or academic tasks. Future studies should examine specific AI-use contexts and patterns to better understand how AI engagement relates to students' career development.

Fourth, the cross-sectional design limits conclusions regarding temporal and causal relationships among AI literacy, AI anxiety, and career optimism. The findings indicate associations among these variables, and reverse relationships are also possible. For example, students with higher career optimism may be more motivated to explore emerging technologies and develop higher AI literacy. In addition, the final regression model explained 33.0% of the variance in career optimism, indicating that substantial variance remains unexplained and that other factors, such as artistic self-efficacy, professional identity, family background, perceptions of the music industry, and institutional support, may contribute to students' career expectations. Longitudinal and mixed-methods studies are needed to further clarify these relationships.

## 5 Conclusion

Among university music students, the findings indicate that AI-related competence and perceived technological threat can coexist. Students with higher perceived AI literacy may still experience substantial AI-related concerns. Higher AI literacy was associated with greater career optimism, whereas higher AI anxiety was associated with lower career optimism, and both associations remained significant after adjustment for gender, year of study, music specialization, prior AI training, and frequency of AI use. These findings suggest that AI-related competence and psychological responses to technological change are important psychological factors related to career expectations among music students. These findings may provide insights for AI education in music institutions, particularly regarding students' understanding of AI capabilities and limitations, evaluation of human expertise, and responses to changing career demands. Future longitudinal and intervention studies are needed to examine temporal relationships and potential mechanisms among AI literacy, AI anxiety, and career optimism.

## Data Availability

The original contributions presented in the study are included in the article/supplementary material, further inquiries can be directed to the corresponding author.
